# Effects of glucagon-like peptide-1 receptor agonists on liver-related and cardiovascular mortality in patients with type 2 diabetes

**DOI:** 10.1186/s12916-023-03228-4

**Published:** 2024-01-04

**Authors:** Fu-Shun Yen, Ming-Chih Hou, James Cheng-Chung Wei, Ying-Hsiu Shih, Chii-Min Hwu, Chih-Cheng Hsu

**Affiliations:** 1 Dr. Yen’s Clinic, No. 15, Shanying Road, Gueishan District, Taoyuan, Taiwan; 2 Division of Gastroenterology and Hepatology, Department of Medicine, Taipei Veterans General Hospital, Taipei, Taiwan; 3 Institute of Clinical Medicine, School of Medicine, National Yang-Ming Chiao Tung University, Taipei, Taiwan; 4 Department of Allergy, Immunology & Rheumatology, Chung Shan Medical University Hospital, Taichung, Taiwan; 5 Institute of Medicine, Chung Shan Medical University, Taichung, Taiwan; 6 Graduate Institute of Integrated Medicine, China Medical University, Taichung, Taiwan; 7 Management Office for Health Data, China Medical University Hospital, Taichung, Taiwan; 8 College of Medicine, China Medical University, Taichung City, Taiwan; 9 Section of Endocrinology and Metabolism, Department of Medicine, Taipei Veterans General Hospital, Taipei, Taiwan; 10 Institute of Population Health Sciences, National Health Research Institutes, Zhunan, Miaoli County Taiwan; 11 Department of Health Services Administration, China Medical University, Taichung, Taiwan; 12 Department of Family Medicine, Min-Sheng General Hospital, Taoyuan, Taiwan; 13 National Center for Geriatrics and Welfare Research, National Health Research Institutes, Yunlin, Taiwan

**Keywords:** All-cause mortality, Liver-related mortality, Cardiovascular mortality, Liver cirrhosis, Hepatic failure

## Abstract

**Background:**

Patients with type 2 diabetes (T2D) tend to have nonalcoholic fatty liver disease (NAFLD) with poorer prognosis. We performed this research to compare the risks of cardiovascular diseases, cirrhosis, liver-related mortality, and cardiovascular mortality between glucagon-like peptide-1 receptor agonist (GLP-1 RA) use and no-use in patients with T2D without viral hepatitis.

**Methods:**

From January 1, 2008, to December 31, 2018, we used propensity-score matching to identify 31,183 pairs of GLP-1 RA users and nonusers from Taiwan’s National Health Insurance Research Database. Multivariable-adjusted Cox proportional hazards models were used to examine the outcomes between the study and control groups.

**Results:**

The median (Q1, Q3) follow-up time for GLP-1 RA users and nonusers were 2.19 (1.35, 3.52) and 2.14 (1.19, 3.68) years, respectively. The all-cause mortality incidence rate was 5.67 and 13.06 per 1000 person-years for GLP-1 RA users and nonusers, respectively. Multivariable-adjusted analysis showed that GLP-1 RA use had significantly lower risks of all-cause mortality (aHR 0.48, 95%CI 0.43–0.53), cardiovascular events (aHR 0.92, 95%CI 0.86–0.99), cardiovascular death (aHR 0.57, 95%CI 0.45–0.72), and liver-related death (aHR 0.32, 95%CI 0.13–0.75). However, there was no significant difference in the risk of liver cirrhosis development, hepatic failure, and hepatocellular carcinoma compared to GLP-1 RA no-use.

**Conclusions:**

This nationwide cohort study showed that GLP-1 RA use was associated with a significantly lower risk of all-cause mortality, cardiovascular events, and cardiovascular death in patients with T2D among Taiwan population. More prospective studies are warranted to verify our results.

**Supplementary Information:**

The online version contains supplementary material available at 10.1186/s12916-023-03228-4.

## Background

Nonalcoholic fatty liver disease (NAFLD) is the accumulation of hepatic fat (≧5%) without much alcohol consumption (< 20 g/day) [1]. In the past 2–3 decades, NAFLD has evolved from an obscure liver disease to the most common chronic liver disease [2]. About 25% of the adult population has NAFLD worldwide [3], and the proportion of NAFLD can be more than 50% in persons with obesity or type 2 diabetes (T2D) [3]. Patients with NAFLD have 2.2 folds increased risk of type 2 diabetes development [4]. In patients with T2D, due to insulin resistance, there is overflow of free fatty acid (FFA) to the liver; the hepatocytes also have increased de novo lipogenesis, which can be re-esterified to triglyceride and accumulated in the liver, resulting in hepatic steatosis [5]. Therefore, the global prevalence of NAFLD in patients with T2D is about 55–70% [6]. From 2009 to 2019, the prevalence of NAFLD in Taiwan increased from 4.49 million to 5.43 million. The number of deaths attributed to NAFLD also increased from 900 to 1,008. Meanwhile, the prevalence of diabetes mellitus in Taiwan increased from 1.39 million in 2009 to 1.9 million in 2019, and the number of deaths attributed to diabetes mellitus increased from 8,892 in 2009 to 11,323 in 2019 [7]. Additionally, glucotoxicity and lipotoxicity in patients with coexisting T2D and NAFLD produce a significant increase in reactive oxygen species, oxidative stress, and mitochondrial dysfunction [4]. This makes the NAFLD in patients with T2D be prone to nonalcoholic steatohepatitis (NASH), fibrosis, cirrhosis, liver cancers, liver-related mortality, and cardiovascular mortality [4].

In response to meal intake, intestinal L-cells produce glucagon-like peptide-1 that stimulates the pancreas to secrete insulin and decrease postprandial blood glucose [8, 9]. Glucagon-like peptide-1 receptor agonists (GLP-1 RAs) were approved for the treatment of type 2 diabetes in 2005 [9]. Large randomized clinical trials have demonstrated that GLP-1 RA can effectively reduce blood glucose, body weight, and the risk of cardiovascular diseases [9]. Animal studies reveal that GLP-1 RAs can improve insulin sensitivity and reduce oxidative stress, hepatic steatosis, and fibrosis [10]. A randomized controlled trial in 52 people with NASH showed that liraglutide improved liver function, had a significantly 30% higher rate of NASH resolution and a 27% lower risk of progression of liver fibrosis [11]. A randomized controlled trial in 320 people with NASH showed that subcutaneous semaglutide led to a significantly higher proportion of people with NASH resolution (23–42%) than placebo, but with no statistically significant difference (10%, *p* = 0.48) in improvement in fibrosis stage [12]. These studies demonstrated some promising results in stopping NAS progression, but they did not further investigate the preventive effects of GLP-1 RA on cirrhosis. Moreover, there are currently no approved medications for treating NAFLD and NASH [1, 2]. There are no studies evaluating GLP-1 RA in relation to cirrhosis and liver-related mortality in patients with T2D. Patients with T2D have an increased risk of cardiovascular disease and cardiovascular mortality, which has subsequently become the leading cause of death in patients with T2D for decades [13]. Therefore, we performed the data analysis from this prospective cohort study in Taiwan to compare the risks of cardiovascular diseases, liver cirrhosis, liver-related mortality, and cardiovascular mortality between GLP-1 RA use and no-use in patients with T2D (excluding those with viral hepatitis or alcohol-related disorders).

## Methods

### Study population and data source

The data source of this research is the full population file of Taiwan’s National Health Insurance Research Database (NHIRD). The features of the NHIRD have been described in our previous study [14]. The diagnosis of diseases in the NHIRD is based on the International Classification of Diseases Ninth/Tenth Revision, Clinical Modification (ICD-9/10-CM). The NHIRD has links to the National Death Registry dataset for death information. All patient and health care information was encrypted before release to protect the privacy of individuals. The Research Ethics Committee of China Medical University and Hospital (CMUH104-REC2-115-CR4) approved this study and waived informed consent from patients.

### Study design and procedures

We identified patients diagnosed with type 2 diabetes from the National Health Insurance Database between January 1, 2008, and December 31, 2018, who received follow-ups till December 31, 2019 (Additional file 1: Fig. S1). T2D was diagnosed according to the ICD codes (Additional file 1: Table S1) with antidiabetic drug use and at least two outpatient visits or one hospitalization within one year for T2D. The algorithm for defining T2D using ICD codes was validated as 74.6% accurate [15]. Exclusion criteria were as follows: missing age or gender data, age below 18 or above 80 years, diagnosis of type 1 diabetes, hepatitis B virus infection, hepatitis C virus infection, alcohol-related disorders, dialysis, liver cirrhosis, esophageal varices with bleeding, ascites, hepatic encephalopathy, jaundice, hepatic failure, hepatocellular carcinoma (HCC), and liver transplant before the index date. The study also excluded patients who died or were lost to follow-up and diagnosed with HCC within 6 months of the index date to avoid latent morbidity or mortality.

We defined patients who had received GLP-1 RAs after T2D diagnosis as GLP-1 RA users and those who had never received GLP-1 RAs during the study period as nonusers. The first date of GLP-1 RA use was set as the index date of the study group. We recorded the index date for the control cases with the same time interval from T2D diagnosis to the index date of the study group. Since GLP-1 RAs were launched in Taiwan in 2011, the index dates for the study and control groups were recorded after 2011. Some clinically relevant variates assessed were as follows: age, sex, family income, obesity, smoking, hypertension, dyslipidemia, stroke, coronary artery disease, heart failure, chronic kidney disease, chronic obstructive pulmonary disease (COPD, diagnosed one year before the index date), and number of oral antidiabetic drugs, insulin, antihypertensive drugs, aspirin, statin, fibrates use (drug use one year prior to index date), and duration of diabetes. We also counted the scores of the Charlson Comorbidity Index (CCI) [16] and Diabetes Complications Severity Index (DCSI) [17] to evaluate the disease burden and diabetes complications in these patients.

### The outcomes of interest

This study compared the use or no-use of GLP-1 RAs in the following outcomes: liver cirrhosis development [18], hepatic failure (coagulopathy, hepatic coma, with or without other organ failures) [19], hepatocellular carcinoma, major adverse cardiovascular events (MACE, a composite outcome of admitted stroke, coronary artery disease, and heart failure), liver-related death (death due to liver cirrhosis, decompensated cirrhosis, hepatic failure, and HCC), cardiovascular death, and all-cause mortality (the confirmation and cause of death were from the link with the National Death Registry). For the outcomes of interest, we followed up with the patients until the occurrence of outcomes, death, or the end of the study period on December 31, 2019, whichever appeared first.

### Statistical analysis

We performed 1:1 propensity score matching to match and balance the variation in the study and control groups [20]. Non-parsimonious multivariable logistic regression was used to estimate the propensity score for every person who received GLP-1 RAs. The GLP-1 RA use was treated as a dependent variable, and 37 critical variables (including age, sex, income, obesity, smoking, comorbidities, medications and duration of diabetes) were used as independent variables (Table 1). We used greedy nearest-neighbor matching to perform optimal matching and matched the control group without replacement. The nearest-neighbor algorithm was used to identify matched pairs with a width of less than 0.001. We assumed a standardized mean difference (SMD) of less than 0.1 as a negligible difference between the study and control groups.

**Table 1:** Comparison of baseline characteristics in patients with T2D with and without GLP-1 RA

Variables	Pre-matched patients		Pre-matched patients		SMD	Post-matched patients		Post-matched patients		SMD
	**without GLP-1 RA**		**with GLP-1 RA**			**without GLP-1 RA**		**with GLP-1 RA**		
	**(*N* = 1538,337)**		**(*N* = 31216)**			**(*N* = 31156)**		**(*N* = 31156)**		
	**n**	**%**	**n**	**%**		**n**	**%**	**n**	**%**	
Sex										
female	783862	50.96	16770	53.72	0.055	16664	53.49	16728	53.69	0.004
male	754475	49.04	14446	46.28	0.055	14492	46.51	14428	46.31	0.004
Age										
18–40	127181	8.27	5783	18.53	0.305	5613	18.02	5744	18.44	0.011
41–60	667332	43.38	15557	49.84	0.130	15567	49.96	15536	49.87	0.002
61–80	743824	48.35	9876	31.64	0.346	9976	32.02	9876	31.70	0.007
mean, (SD)^a^	58.90	12.11	53.09	12.97	0.463	53.35	12.92	53.12	12.96	0.018
Income, New Taiwan Dollars										
< 22000	361136	23.48	6195	19.85	0.088	6151	19.74	6189	19.86	0.003
22000–44999	891505	57.95	18393	58.92	0.020	18399	59.05	18359	58.93	0.003
> 44999	285,696	18.57	6628	21.23	0.067	6606	21.20	6608	21.21	< 0.001
Comorbidities										
Obesity	45042	2.93	3711	11.89	0.347	3492	11.21	3670	11.78	0.018
Smoking	41955	2.73	1348	4.32	0.086	1361	4.37	1344	4.31	0.003
Hypertension	976049	63.45	22009	70.51	0.150	22073	70.85	21970	70.52	0.007
Dyslipidemia	1,098,806	71.43	26660	85.40	0.345	26756	85.88	26602	85.38	0.014
Stroke	79714	5.18	1448	4.64	0.025	1465	4.70	1448	4.65	0.003
Coronary artery disease	272876	17.74	5204	16.67	0.028	5233	16.80	5200	16.69	0.003
Heart failure	53934	3.51	1225	3.92	0.022	1164	3.74	1222	3.92	0.010
Chronic kidney disease	199447	12.97	6414	20.55	0.204	6481	20.80	6398	20.54	0.007
COPD	364499	23.69	7649	24.50	0.019	7515	24.12	7627	24.48	0.008
CCI										
1	1364298	88.69	25273	80.96	0.217	25128	80.65	25225	80.96	0.008
2–3	151095	9.82	4789	15.34	0.167	4842	15.54	4779	15.34	0.006
> 3	22944	1.49	1154	3.70	0.139	1186	3.81	1152	3.70	0.006
DCSI										
0	606665	39.44	6475	20.74	0.416	6436	20.66	6470	20.77	0.003
1	336418	21.87	6008	19.25	0.065	5921	19.00	6000	19.26	0.006
≥ 2	595254	38.69	18733	60.01	0.436	18799	60.34	18686	59.98	0.007
Medication										
Metformin	993488	64.58	30488	97.67	0.933	30619	98.28	30428	97.66	0.043
Sulfonylurea	699012	45.44	27292	87.43	0.993	27390	87.91	27233	87.41	0.015
Meglitinides	114479	7.44	7562	24.22	0.472	7516	24.12	7537	24.19	0.002
Thiazolidinedione	210669	13.69	16148	51.73	0.887	16280	52.25	16103	51.69	0.011
DPP-4 inhibitors	337,587	21.94	25403	81.38	1.479	25880	83.07	25343	81.34	0.045
SGLT2 inhibitors	33373	2.17	7369	23.61	0.675	6736	21.62	7315	23.48	0.044
Insulin	247598	16.10	20628	66.08	1.180	20560	65.99	20568	66.02	0.001
ACEI/ARB	805284	52.35	21328	68.32	0.331	21318	68.42	21278	68.30	0.003
β-blockers	373613	24.29	6896	22.09	0.052	6820	21.89	6885	22.10	0.005
Calcium-channel blockers	797882	51.87	16515	52.91	0.021	16648	53.43	16492	52.93	0.010
Diuretics	447044	29.06	11302	36.21	0.153	11315	36.32	11281	36.21	0.002
Statin	838452	54.50	24265	77.73	0.506	24345	78.14	24211	77.71	0.010
Fibrates	337082	21.91	10790	34.57	0.284	10632	34.13	10766	34.56	0.009
Aspirin	460946	29.96	11425	36.60	0.141	11453	36.76	11409	36.62	0.003
Number of oral antidiabetic drugs										
1	763995	49.66	1027	3.29	1.235	749	2.40	1027	3.30	0.054
2–3	547930	35.62	6658	21.33	0.321	6551	21.03	6658	21.37	0.008
> 3	226412	14.72	23531	75.38	1.538	23856	76.57	23471	75.33	0.029
Duration of diabetes, years										
Mean, (SD)	3.88	2.89	6.95	3.20	1.006	6.90	3.06	6.94	3.20	0.014

*T2D* type 2 diabetes, *GLP-1 RAs* glucagon-like peptide-1 receptor agonists, *COPD* chronic obstructive pulmonary disease, *CCI* Charlson Comorbidity Index, *DCSI* Diabetes Complications Severity Index, *DPP-4* dipeptidyl peptidase-4, *SGLT2* sodium-glucose cotransporter-2, *ACEI* angiotensin-converting enzyme inhibitor, *ARB* angiotensin receptor blocker. Data shown as n (%) or mean ± SD

^a^: Student’s t-test. SMD: standardized mean difference. A standardized mean difference of 0.1 or less indicates a negligible difference

Crude and multivariate-adjusted Cox proportional hazards models were used to compare the endpoints between GLP-1 RA users and nonusers. We presented the results as hazard ratios (HR) and 95% confidence interval (CI). The Schoenfeld residuals and complementary log–log plots were used to check the proportional-hazards assumption. We used a stepwise approach to adjust for the variables in the Cox models, as follows: Model 1: adjusted for sex and age; Model 2: adjusted for sex, age, income, and obesity; Model 3: adjusted for sex, age, income, obesity, and comorbidities; Model 4: adjusted for sex, age, income, obesity comorbidities, medications and duration of diabetes as shown in Table 1. Kaplan–Meier method was used to describe the cumulative incidence of outcomes between GLP-1 RA use and no-use over the follow-up time. We performed the subgroup analysis, including the subgroups of sex, age, comorbidities, CCI, DCSI, and medications, of GLP-1 RA use versus no use in the outcomes of all-cause death, cardiovascular diseases, cardiovascular death, and liver-related death. We also performed dose–response analysis on the cumulative duration of < 182, 182–364, > 364 days of GLP-1 RA use versus no use in the outcomes of all-cause death, cardiovascular diseases, cardiovascular death, and liver-related death.

We considered the two-tailed *p*-value < 0.05 statistically significant and used SAS version 9.4 and Stata SE version 11.0 for analysis.

## Results

From January 1, 2008, to December 31, 2018, we identified 3,432,732 newly diagnosed T2D patients from our NHIRD (Additional file 1: Fig. S1). We used a 1-to-1 propensity score matching method to find 31,183 pairs of GLP-1 RA nonusers and users. The total follow-up time for the study is 167,619 person-years. The median (Q1, Q3) follow-up time for GLP-1 RA users and nonusers were 2.19 (1.35, 3.52) and 2.14 (1.19, 3.68) years, respectively.

Clinically-related variables were all well-matched between the study and control groups (Table 1).

Among the matched cohorts of patients with T2D, 1093 (3.51%) GLP-1 RA nonusers and 480 (1.54%) users died during the follow-up period; the incidence rates of all-cause mortality were 13.06 and 5.67 per 1000 person-years, respectively (Table 2). The multivariable-adjusted models showed that GLP-1 RA use was associated with a significantly lower risk of all-cause mortality (Model 1,2,3,4 aHRs are 0.46,0.46,0.46,0.48) than no-use of GLP-1 RAs (Table 2). Compared with GLP-1 RA no-use, GLP-1 RA use was also associated with a significantly reduced risk of liver-related death (Model 1,2,3,4 aHRs are 0.36, 0.35, 0.35, 0.32), major adverse cardiovascular events (Model 1,2,3,4 aHRs are 0.92, 0.91, 0.92, 0.92), and cardiovascular death (Model 1,2,3,4 aHRs are 0.55, 0.54, 0.55, 0.57), but without significant difference in the risks of cirrhosis development (aHR 1.10, 95%CI 0.88–1.37), hepatic failure (aHR 0.92, 95%CI 0.66–1.30), and hepatocellular carcinoma (aHR 0.91, 95%CI 0.59–1.40).

**Table 2:** Hazard ratios for outcomes in patients with T2D with and without GLP-1 RAs

**Outcome**	**T2D patients without GLP-1 RA**			**T2D patients with GLP-1 RA**								
	**n**	**PY**	**IR**	**n**	**PY**	**IR**	**cHR**	**aHR**^**1**^	**aHR**^**2**^	**aHR**^**3**^	**aHR**^**4**^	**(95% CI)**
All-cause mortality	1093	83691	13.06	480	84680	5.67	0.43	0.46	0.46	0.46	0.48	(0.43, 0.53)***
MACE	1653	80860	20.44	1486	82211	18.08	0.88	0.92	0.91	0.92	0.92	(0.86, 0.99)*
Cardiovascular death	197	83691	2.35	103	84680	1.22	0.52	0.55	0.54	0.55	0.57	(0.45, 0.72)***
Liver-related death	22	83691	0.26	7	84680	0.08	0.31	0.36	0.35	0.35	0.32	(0.13, 0.75)**
Liver cirrhosis	155	83495	1.86	165	84421	1.95	1.05	1.08	1.08	1.08	1.10	(0.88, 1.37)
Hepatic failure	71	83603	0.85	63	84596	0.74	0.87	0.90	0.89	0.90	0.92	(0.66, 1.30)
Hepatocellular carcinoma	44	83685	0.53	41	84664	0.48	0.93	1.02	1.00	1.01	0.91	(0.59, 1.40)

*T2D* type 2 diabetes, *GLP-1 RAs* glucagon-like peptide-1 receptor agonists, *PY* person-years, *IR* incidence rate, per 1,000 person-years, *cHR* crude hazard ratio, *aHR* adjusted hazard ratio, *MACE* major adverse cardiovascular events; aHR^1^: Adjusted for sex, and age; aHR^2^: Adjusted for sex, age, income, and obesity; aHR^3^: Adjusted for sex, age, income, obesity, and comorbidities; aHR^4^: adjusted for sex, age, income, obesity, comorbidities, medications, and duration of diabetes as shown in Table 1

^*^ *P* < 0.05

^**^ *P* < 0.01

^***^ *P* < 0.001

**Fig. 1:**
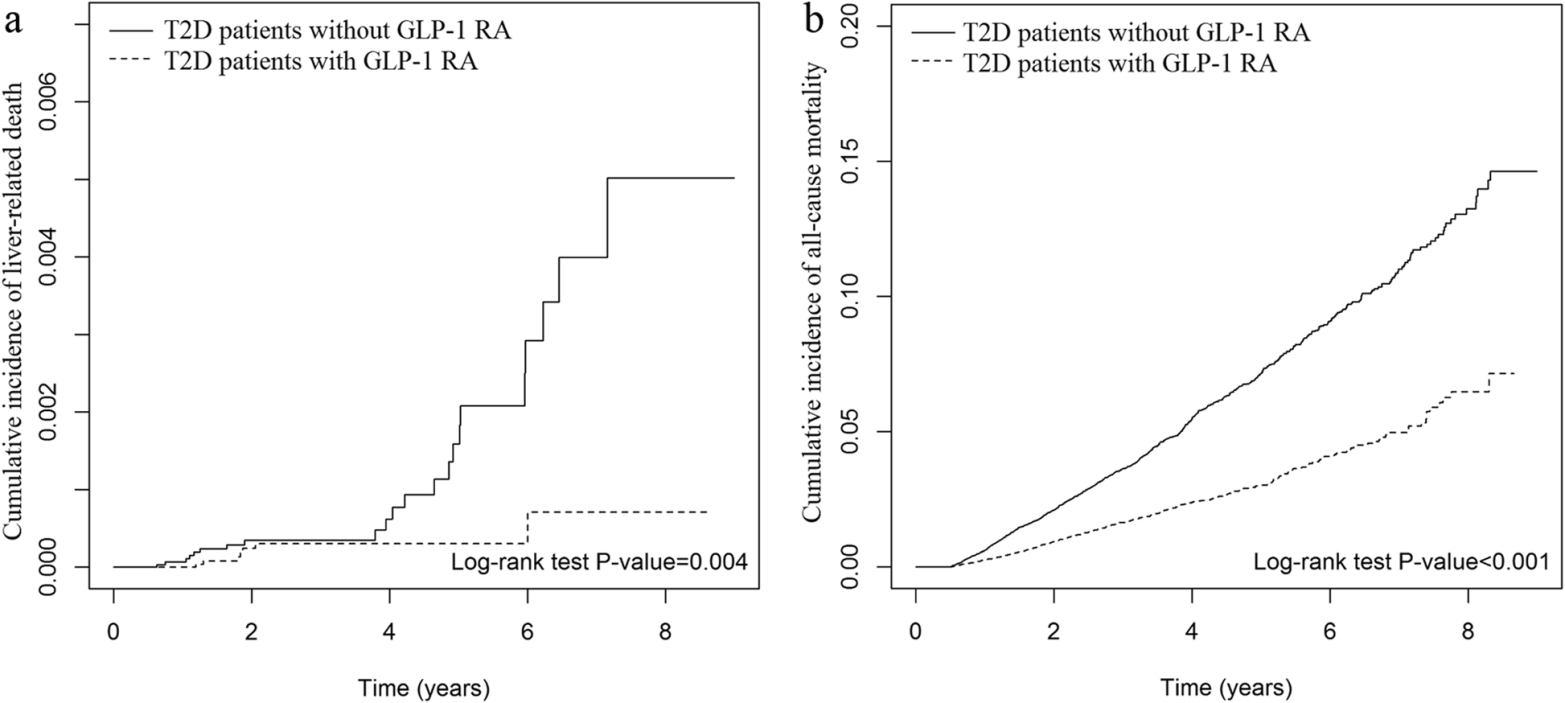
The cumulative incidences of liver-related death (**a**), mortality (**b**) between GLP-1 RA users and nonusers

The Kaplan–Meier method showed that GLP-1 RA use had a significantly lower risk in cumulative incidences of liver-related death (Log-rank test *p* = 0.004), all-cause death (Log-rank test *p* < 0.001; Fig. 1), cardiovascular events (Log-rank test *p* < 0.001), and cardiovascular death (Log-rank test, *p* < 0.001; Additional file 1: Fig. S2) than GLP-1 RA no-use.

Subgroup analysis of GLP-1 RA use vs. no-use in the risks of cardiovascular disease, cardiovascular mortality, and all-cause mortality showed that GLP-1 RA use was associated with a lower risk in the subgroups of gender, age, comorbidities, and drug use (Additional file 1: Table S2-S4). However, in the subgroups for obesity [aHR 1.08, 95%CI 0.85–1.35) and SGLT2 inhibitors use (aHR 1.05, 95%CI 0.82–1.35), GLP-1 RAs were associated with a non-significantly higher risk of cardiovascular disease (Additional file 1: Table S4). The potential reason for this could be that these two subgroups have smaller sample sizes, resulting in a lower incidence rate of cardiovascular events, which in turn leads to less stable statistical results. Compared with no-use of GLP-1 RAs, GLP-1 RA use had a significantly lower risk of liver-related death in the subgroups of sex (female, male, *p* < 0.05) and age (< 70, *p* = 0.0015) (Additional file 1: Table S5).

Compared to no use of GLP-1 RAs, the cumulative duration of < 182, 182–364, > 364 days of GLP-1 RA use showed a significantly reduced risk of all-cause death with a significant p-value for trend (*p* < 0.0001). Cumulative duration of 182–364, > 364 days of GLP-1 RA use exhibited a significantly lower risk of major adverse cardiovascular events and cardiovascular death with a with a significant *p*-value for trend (*p* < 0.0001) of cardiovascular death. A cumulative duration of ≧90 days of GLP-1 RA use showed a significantly lower risk of liver-related death with a significant *p*-value for trend (*p* = 0.0045; Table 3).

**Table 3:** Hazard ratio of outcomes stratified by the cumulative duration of GLP-1 RAs

Variables	All-cause mortality						
	n	PY	IR	cHR	(95% CI)	aHR^†^	(95% CI)
Non-use of GLP-1 RAs	1093	83691	13.06	1.00	(reference)	1.00	(reference)
Cumulative duration of GLP-1 RAs (days)							
> 182	308	33875	9.09	0.74	(0.65, 0.84)***	0.79	(0.69, 0.90)***
182–364	101	21978	4.60	0.38	(0.31, 0.46)***	0.44	(0.36, 0.54)***
> 364	71	28827	2.46	0.17	(0.13, 0.21)***	0.19	(0.15, 0.24)***
P for trend						< 0.0001	
	Liver-related death						
Variables	n	PY	IR	cHR	(95% CI)	aHR^†^	(95% CI)
Non-use of GLP-1 RAs	22	83691	0.26	1.00	(reference)	1.00	(reference)
Cumulative duration of GLP-1 RAs (days)							
< 90	3	20886	0.14	0.56	(0.17, 1.88)	0.50	(0.15, 1.69)
≧90	4	63794	0.06	0.23	(0.08, 0.68)**	0.25	(0.09, 0.73)*
P for trend						0.0045	
Variables	Cardiovascular mortality						
	n	PY	IR	cHR	(95% CI)	aHR^†^	(95% CI)
Non-use of GLP-1 RAs	197	83691	2.35	1.00	(reference)	1.00	(reference)
Cumulative duration of GLP-1 RAs (days)							
> 182	67	33875	1.98	0.90	(0.68, 1.19)	0.97	(0.73, 1.28)
182–364	24	21978	1.09	0.50	(0.33, 0.76)**	0.59	(0.38, 0.90)*
> 364	12	28827	0.42	0.16	(0.09, 0.28)***	0.17	(0.09, 0.31)***
P for trend						< 0.0001	
Variables	Cardiovascular events						
	n	PY	IR	cHR	(95% CI)	aHR^†^	(95% CI)
Non-GLP-1 RA drug days	1653	80860	20.44	1.00	(reference)	1.00	(reference)
Cumulative duration of GLP-1 RAs (days)							
> 182	710	32784	21.66	1.12	(1.02, 1.22)*	1.16	(1.06, 1.26)**
182–364	330	21484	15.36	0.77	(0.69, 0.87)***	0.82	(0.73, 0.93)**
> 364	446	27943	15.96	0.72	(0.65, 0.80)***	0.75	(0.68, 0.83)***
P for trend						0.6991	

*GLP-1 RAs* glucagon-like peptide-1 receptor agonists, *PY* person-years, *IR* incidence rate per 1,000 person-years, *cHR* crude hazard ratio, *aHR* adjusted hazard ratio, *MACE* major adverse cardiovascular events

^*^ *p*-value < 0.05

^**^p < 0.01

^***^ *p* < 0.001

^†^: adjusted by sex, age, comorbidities, medication, CCI, DCSI, and duration of T2D, as shown in Table 1

## Discussion

This study showed that GLP-1 RA use was associated with a significantly lower risk of all-cause mortality, cardiovascular events, cardiovascular and liver-related mortality. However, there was no significant difference in the risk of cirrhosis development, hepatic failure, and hepatocellular carcinoma in patients with T2D and without viral hepatitis. Compared with the no-use of GLP-1 RAs, a longer cumulative duration of GLP-1RA use had a lower risk of liver-related and cardiovascular mortality, all-cause mortality, and cardiovascular events. Moreover, subgroups of age, gender, comorbidities, and medications showed reduced risks of cardiovascular events and cardiovascular and overall mortality from GLP-1RAs.

NAFLD is considered the most common cause of cryptogenic cirrhosis; however, there are no adequate preventive measures [21]. One meta-analysis showed that liraglutide could significantly improve alanine transaminase (ALT) levels more than placebo in patients with T2D [22]. A randomized controlled study on liraglutide in 52 persons with NASH (with or without T2D) showed that liraglutide had a significantly higher rate of NASH resolution and lower risk in the progression of liver fibrosis [11]. A randomized controlled trial of semaglutide in 320 persons with NASH (with or without T2D) showed that subcutaneous semaglutide led to a significantly higher proportion of persons with the resolution of NASH than the placebo group, with no statistically significant difference in the improvement of fibrosis stage [12]. The post hoc analysis of phase 2 of the tirzepatide trial showed that a higher dose of this novel dual glucose-dependent insulinotropic polypeptide (GIP) and GLP-1 receptor agonist could significantly decrease the level of NASH-related biomarkers and increase adiponectin level in patients with T2D [23]. These studies suggest that GLP-1RAs may improve liver function and insulin sensitivity, reduce liver fat and hepatic inflammation, and increase the resolution of NASH; but about the mitigation of hepatic fibrosis and prevention of liver cirrhosis are unclear. Our study showed that GLP-1 RA use in patients with T2D without viral hepatitis exhibited no significant difference in preventing liver cirrhosis development compared to GLP-1 RA no-use.

Patients with NASH, especially those with fibrosis, constitute a high-risk group for HCC, and the number of HCCs caused by NAFLD is increasing [1, 24]. Furthermore, diabetes can increase the risk of liver cancers in patients with NAFLD [1, 4]. Large-scale randomized control trials have not shown an association between GLP-1 RAs and liver cancers [9]. Our large-scale study also showed no significant difference in the risk of incident HCC between GLP-1 RA use and no-use in patients with T2D.

GLP-1 RAs are not mainly eliminated by hepatic metabolism [9]. No clinical study has shown prominent hepatotoxicity with GLP-1 RA use [25]. Our study also showed no significant association between GLP-1 RA use and hepatic failure. However, it showed that GLP-1 RA use was associated with a lower risk of liver-related mortality. Simon et al. conducted a cohort study showing that GLP-1 RA use was associated with a lower risk of cirrhotic decompensation than DPP-4 inhibitors or sulfonylureas in patients with liver cirrhosis [26]. GLP-1 RA use might reduce the risk of hepatic complications or cirrhotic decompensation in our patients after enrolment and reduce the risk of liver-related death. However, because the incidence of liver-related death, hepatic failure, and hepatocellular carcinoma is low in this study, the results regarding liver-related death, hepatic failure, and hepatocellular carcinoma should be interpreted with extreme caution.

Cardiovascular disease is the major complication and cause of death in patients with T2D or NAFLD [1, 27]. In patients with NAFLD, higher inflammatory scores have a higher risk of cardiovascular disease [24, 27]. The cardiovascular outcome trials of GLP-1 RAs demonstrated that GLP-1 RAs could significantly reduce the risk of major adverse cardiovascular events and cardiovascular mortality in patients with T2D [9]. Our study also revealed that GLP-1 RA use was associated with a significantly lower risk of cardiovascular diseases and cardiovascular mortality than GLP-1 RA no-use in patients with T2D and without viral hepatitis. GLP-1 RAs may reduce the risk of cardiovascular morbidity and mortality by improving dyslipidemia, lowering blood pressure, and modifying the process of atherosclerosis [10, 27, 28].

Nonalcoholic fatty liver disease, especially nonalcoholic steatohepatitis, has a higher liver-related and overall mortality risk than the general population [1, 3]. Randomized studies have shown that GLP-1 RAs could reduce the risk of all-cause mortality compared to a placebo in patients with T2D [28]. Our results also demonstrated that GLP-1 RA use was associated with a lower risk of all-cause mortality compared to no-use of GLP-1 RAs in patients with T2D and without viral hepatitis, which may be attributable to the reduced risks of cardiovascular events, and liver-related and cardiovascular mortality with GLP-1 RA use in this study.

This study has some disadvantages. First, the National Health Insurance database lacked details on smoking habits, diet, physical activity, and family history, which may have affected the results. We tried to balance the baseline characteristics of the study and control groups to increase their comparability. Second, this database lacked details on hemoglobin A1C, glucose, lipid indicators (TC, TG, LDL-c, HDL-c), liver function (ALT, AST), kidney function (CCr, Bun), cardiac function (TnT), imaging, and histopathology results. This limitation prevented us from evaluating the diabetes status and accurately diagnosing and staging NAFLD, NASH, liver fibrosis, and cirrhosis. We used the CCI, DCSI scores, insulin, and the number of oral antidiabetic drugs to evaluate T2D severity. We used the diagnosis of T2D and excluded patients with hepatitis B, hepatitis C infection, and alcohol-related disorders to create a representative cohort of patients with T2D and NAFLD. Third, the participants in this study were mainly of Chinese ethnicity. Therefore, the results may not apply to other ethnic groups. However, our results may provide some important information about the Oriental population. Finally, a cohort study is usually subject to unknown or unobserved confounding factors; therefore, randomized controlled trials are recommended to confirm our results.

## Conclusions

Our research showed that GLP-1 RAs could not prevent the development of liver cirrhosis. However, it could reduce the risks of cardiovascular disease, cardiovascular, and overall mortality in patients with type 2 diabetes without viral hepatitis among Taiwan population. GLP-1 RAs may be suitable for patients with type 2 diabetes coexisting with NAFLD. However, more prospective studies and randomized controlled trials are warranted to verify the findings.

### Supplementary Information

**Additional file 1: Table S1.** Diseases and related ICD-9-CM, ICD-10-CM codes. **Table S2.** The risk of all-cause death for T2D patients with and without GLP-1 RA stratified by variables. **Table S3.** The risk of cardiovascular death for T2D patients with and without GLP-1 RA stratified by variables. **Table S4.** The risk of cardiovascular events for T2D patients with and without GLP-1 RA stratified by variables. **Table S5.** The risk of liver-related death for T2D patients with and without GLP-1 RA stratified by variables. **Fig. S1.** Flowchart of patient selection in this study. **Fig. S2.** The cumulative incidences of major adverse cardiovascular events (MACE, a), cardiovascular death (b), between GLP-1 RA users and nonusers in persons with T2D.

## Data Availability

Data of this study are available from the National Health Insurance Research Database (NHIRD) published by Taiwan National Health Insurance (NHI) Administration. The data utilized in this study cannot be made available in the paper, the supplemental files, or in a public repository due to the ‘‘Personal Information Protection Act’’ executed by Taiwan government starting from 2012. Requests for data can be sent as a formal proposal to the NHIRD Office (https://dep.mohw.gov.tw/DOS/cp-2516-3591-113.html) or by email to stsung@mohw.gov.tw.

## Abbreviations

T2D: Type 2 diabetes
GLP-1: RA glucagon-like peptide 1 receptor agonist
NAFLD: Nonalcoholic fatty liver disease
NASH: Nonalcoholic steatohepatitis
HCC: Hepatocellular carcinoma
HBV: Hepatitis B virus
HCV: Hepatitis C virus
SGLT2: Sodium-glucose cotransporter 2
