# Supplementary material for: Effects of glucagon-like peptide-1 receptor agonists on liver-related and cardiovascular mortality in patients with type 2 diabetes
Source: BMC Med. 2024 Jan 4;22:8. doi: 10.1186/s12916-023-03228-4 (PMC10765623; doi:10.1186/s12916-023-03228-4)
Supplement: Supplementary file 1 — Additional file 1: Table S1. Diseases and related ICD-9-CM, ICD-10-CM codes. Table S2. The risk of all-cause death for T2D patients with and without GLP-1 RA stratified by variables. Table S3. The risk of cardiovascular death for T2D patients with and without GLP-1 RA stratified by variables. Table S4. The risk of cardiovascular events for T2D patients with and without GLP-1 RA stratified by variables. Table S5. The risk of liver-related death for T2D patients with and without GLP-1 RA stratified by variables. Fig. S1. Flowchart of patient selection in this study. Fig. S2. The cumulative incidences of major adverse cardiovascular events (MACE, a), cardiovascular death (b), between GLP-1 RA users and nonusers in persons with T2D. [file 12916_2023_3228_MOESM1_ESM.zip › Supplementary information 20231002R3.docx]

**Table S1** Diseases and related ICD-9-CM, ICD-10-CM codes

| **Disease** | **ICD-9-CM codes** | **ICD-10-CM codes** |
| --- | --- | --- |
| Type 2 diabetes | 250.xx, except 250.1x | E11 |
| Type 1 diabetes | 250.1x | E10 |
| Hepatitis B infection | 070.2, 070.3, V02.61 | B16.2, B16.1, B16.0, B16.9, B18.0, B18.1, B19.10, B19.11, Z22.51 |
| Hepatitis C infection | 070.41, 070.44, 070.51, 070.54, 070.70, 070.71, V02.62 | B17.10, B17.11, B19.20, B19.21, B18.2, Z22.52 |
| Alcohol-related disorder | 291, 303, 305.0, 571.0-571.3, V11.3, V79.1 | F10, K70.40, K70.41, K70.9 |
| Liver cirrhosis | 571.5, 571.2, or 571.6 | K70.2, K70.30, K70.31, K74.0, K74.1, K74.2, K74.60, K74.69, K74.3, K74.4, K74.5 |
| Ascites | 789.59, 789.5 | R18.8 |
| Hepatic encephalopathy | 572.2 | K72.90, K72.91 |
| Esophageal varices with bleeding | 456.0, 456.2 | I85.01 |
| Jaundice | 782.4 | R17 |
| Hepatic failure | 570, 572.2, 572.4, 572.8 | K72.00, K72.01, K72.10, K72.11, K72.90, K76.2, K72.90, K72.91, K76.7, K76.81 |
| Liver transplant | V42.7,996.82, procedure code 50.5 | Z94.4, D89.810, D89.811, D89.812, D89.813, T81.40, T81.41, T81.42, T81.43, T81.49, 0FY00Z0, 0FY00Z1, 0FY00Z2 |
| Hepatocellular carcinoma | 155.x | C22 |
| Smoking | 305.1, 649.0, V15.82 | F17.200, F17.201, F17.210, F17.220, F17.221, F17.290, F17.291, Z87.891 |
| Obesity | 278.02, 783.1, V85.2, 278.00, 649.1, V77.8, V85.3, 278.01, 649.2, V45.86, V85.4 | R63.5, E66.09, E66.1, E66.8, E66.9, Z13.89, E66.01, E66.2 |
| Hypertension | 401–405 and A26 | I10, I11, I12, I13, I15, N26.2 |
| Dyslipidemia | 272 | E71.30, E71.31, E71.32, E71.39, E75.21, E75.22, E75.23, E75.24, E75.25, E75.29, E75.3, E75.4, E75.5, E75.6, E77, E78.0, E78.1, E78.2, E78.3, E78.4, E78.5, E78.6, E78.70, E78.71, E78.72, E78.79, E78.8, E78.9 |
| Stroke | 430-437 | G45.0, G45.1, G45.2, G45.3, G45.4, G45.8, G45.9, G46, I60, I61, I62, I63, I65, I66, I67.0, I67.1, I67.2, I67.3, I67.4, I67.5, I67.6, I67.7, I67.8, I67.9, I68, I69 |
| Coronary artery disease | 410-414 | I20, I21, I22, I24, I25.1, I25.2, I25.3, I25.4, I25.5, I25.6, I25.7, I25.81, I25.82, I25.83, I25.84, I25.89, I25.9 |
| Heart failure | 428 | I50 |
| Chronic kidney disease | 250.4x, 403.xx, 404.xx, 585.xx, 586.xx, 581.8x, 791.0x, 593 | E10.2, E10.65, E11.2, E11.65, E13.2, I12, I13, N03, N08, E10.21, E11.21, N05, N06, N07, N14, N15.0, N15.8, N15.9, N16, N17.1, N17.2, N18, N19 |
| Dialysis | V56.0, V56.8, V45.1 | Z49.31, Z49.32, Z99.2 |
| Chronic obstructive pulmonary disease | 491, 492, or 496 | J41, J42, J44, J43, or J44.9 |

**Table S2** The risk of all-cause death for patients with and without GLP-1 RA stratified by variables

| **Variable** | **Non-GLP-1 RA** | | | **GLP-1 RA use** | | | **Crude** | | | **Adjusted†** | | |
| --- | --- | --- | --- | --- | --- | --- | --- | --- | --- | --- | --- | --- |
|  | **n** | **PY** | **IR** | **n** | **PY** | **IR** | **cHR** | **95%CI** | **P-value** | **aHR** | **95%CI** | **P-value** |
| Sex |  |  |  |  |  |  |  |  |  |  |  |  |
| female | 571 | 49050 | 11.64 | 191 | 42387 | 4.51 | 0.42 | (0.36, 0.5) | <0.001 | 0.46 | (0.39, 0.54) | <0.001 |
| male | 613 | 42178 | 14.53 | 196 | 35881 | 5.46 | 0.4 | (0.34, 0.47) | <0.001 | 0.41 | (0.35, 0.49) | <0.001 |
| Age |  |  |  |  |  |  |  |  |  |  |  |  |
| 20-40 | 63 | 15907 | 3.96 | 28 | 16531 | 1.69 | 0.43 | (0.27, 0.67) | <0.001 | 0.4 | (0.26, 0.63) | <0.001 |
| 41-60 | 409 | 46667 | 8.76 | 135 | 40892 | 3.30 | 0.4 | (0.33, 0.49) | <0.001 | 0.41 | (0.33, 0.49) | <0.001 |
| >60 | 712 | 28654 | 24.85 | 224 | 20845 | 10.75 | 0.51 | (0.44, 0.59) | <0.001 | 0.53 | (0.45, 0.61) | <0.001 |
| Comorbidities |  |  |  |  |  |  |  |  |  |  |  |  |
| Obesity |  |  |  |  |  |  |  |  |  |  |  |  |
| No | 1123 | 82198 | 13.66 | 347 | 68207 | 5.09 | 0.4 | (0.36, 0.46) | <0.001 | 0.43 | (0.38, 0.48) | <0.001 |
| Yes | 61 | 9031 | 6.75 | 40 | 10062 | 3.98 | 0.59 | (0.4, 0.88) | 0.0101 | 0.59 | (0.39, 0.89) | 0.0108 |
| Smoking |  |  |  |  |  |  |  |  |  |  |  |  |
| No | 1151 | 88573 | 12.99 | 373 | 75633 | 4.93 | 0.41 | (0.36, 0.46) | <0.001 | 0.43 | (0.39, 0.49) | <0.001 |
| Yes | 33 | 2655 | 12.43 | 14 | 2635 | 5.31 | 0.46 | (0.25, 0.86) | 0.0155 | 0.53 | (0.28, 1.02) | 0.0566 |
| Hypertension |  |  |  |  |  |  |  |  |  |  |  |  |
| No | 176 | 27053 | 6.51 | 48 | 23551 | 2.04 | 0.34 | (0.24, 0.46) | <0.001 | 0.35 | (0.25, 0.48) | <0.001 |
| Yes | 1008 | 64176 | 15.71 | 339 | 54717 | 6.20 | 0.42 | (0.38, 0.48) | <0.001 | 0.45 | (0.4, 0.51) | <0.001 |
| Dyslipidemia |  |  |  |  |  |  |  |  |  |  |  |  |
| No | 224 | 13350 | 16.78 | 68 | 12353 | 5.50 | 0.35 | (0.27, 0.46) | <0.001 | 0.38 | (0.29, 0.5) | <0.001 |
| Yes | 960 | 77879 | 12.33 | 319 | 65915 | 4.84 | 0.42 | (0.37, 0.48) | <0.001 | 0.45 | (0.4, 0.52) | <0.001 |
| Stroke |  |  |  |  |  |  |  |  |  |  |  |  |
| No | 1049 | 87231 | 12.03 | 341 | 75202 | 4.53 | 0.4 | (0.36, 0.46) | <0.001 | 0.43 | (0.38, 0.48) | <0.001 |
| Yes | 135 | 3998 | 33.77 | 46 | 3067 | 15.00 | 0.53 | (0.38, 0.75) | <0.001 | 0.55 | (0.39, 0.77) | <0.001 |
| Coronary artery disease |  |  |  |  |  |  |  |  |  |  |  |  |
| No | 928 | 76469 | 12.14 | 294 | 65927 | 4.46 | 0.39 | (0.34, 0.45) | <0.001 | 0.42 | (0.37, 0.48) | <0.001 |
| Yes | 256 | 14760 | 17.34 | 93 | 12342 | 7.54 | 0.48 | (0.38, 0.62) | <0.001 | 0.5 | (0.39, 0.63) | <0.001 |
| Heart failure |  |  |  |  |  |  |  |  |  |  |  |  |
| No | 1085 | 88075 | 12.32 | 355 | 75473 | 4.70 | 0.41 | (0.36, 0.46) | <0.001 | 0.44 | (0.39, 0.5) | <0.001 |
| Yes | 99 | 3154 | 31.39 | 32 | 2796 | 11.44 | 0.39 | (0.26, 0.59) | <0.001 | 0.42 | (0.28, 0.63) | <0.001 |
| Chronic kidney disease |  |  |  |  |  |  |  |  |  |  |  |  |
| No | 782 | 75430 | 10.37 | 257 | 65367 | 3.93 | 0.41 | (0.36, 0.47) | <0.001 | 0.42 | (0.37, 0.49) | <0.001 |
| Yes | 402 | 15799 | 25.45 | 130 | 12902 | 10.08 | 0.44 | (0.36, 0.54) | <0.001 | 0.47 | (0.38, 0.57) | <0.001 |
| COPD |  |  |  |  |  |  |  |  |  |  |  |  |
| No | 876 | 71013 | 12.34 | 271 | 60285 | 4.50 | 0.39 | (0.34, 0.45) | <0.001 | 0.42 | (0.37, 0.48) | <0.001 |
| Yes | 308 | 20215 | 15.24 | 116 | 17983 | 6.45 | 0.47 | (0.38, 0.58) | <0.001 | 0.49 | (0.39, 0.61) | <0.001 |
| CCI |  |  |  |  |  |  |  |  |  |  |  |  |
| 1 | 901 | 80468 | 11.20 | 271 | 68459 | 3.96 | 0.38 | (0.33, 0.44) | <0.001 | 0.41 | (0.36, 0.47) | <0.001 |
| 2-3 | 218 | 9287 | 23.47 | 87 | 8471 | 10.27 | 0.47 | (0.37, 0.61) | <0.001 | 0.51 | (0.4, 0.66) | <0.001 |
| >3 | 65 | 1474 | 44.10 | 29 | 1339 | 21.66 | 0.52 | (0.34, 0.82) | 0.0043 | 0.5 | (0.32, 0.79) | 0.0031 |
| DCSI |  |  |  |  |  |  |  |  |  |  |  |  |
| 0 | 122 | 20509 | 5.95 | 45 | 18343 | 2.45 | 0.44 | (0.31, 0.62) | <0.001 | 0.42 | (0.3, 0.6) | <0.001 |
| 1 | 151 | 18529 | 8.15 | 51 | 16352 | 3.12 | 0.42 | (0.3, 0.57) | <0.001 | 0.42 | (0.3, 0.57) | <0.001 |
| ≥2 | 911 | 52190 | 17.46 | 291 | 43574 | 6.68 | 0.42 | (0.37, 0.48) | <0.001 | 0.44 | (0.39, 0.5) | <0.001 |
| Number of oral antidiabetic drugs | |  |  |  |  |  |  |  |  |  |  |  |
| 1 | 9 | 2831 | 3.18 | 7 | 2354 | 2.97 | 1.47 | (0.5, 4.26) | 0.4834 | 1.79 | (0.51, 6.28) | 0.3626 |
| 2-3 | 166 | 20604 | 8.06 | 68 | 18522 | 3.67 | 0.5 | (0.37, 0.66) | <0.001 | 0.48 | (0.36, 0.64) | <0.001 |
| >3 | 1009 | 67793 | 14.88 | 312 | 57392 | 5.44 | 0.39 | (0.35, 0.45) | <0.001 | 0.42 | (0.37, 0.48) | <0.001 |
| Medication |  |  |  |  |  |  |  |  |  |  |  |  |
| Metformin |  |  |  |  |  |  |  |  |  |  |  |  |
| No | 18 | 1838 | 9.79 | 10 | 1655 | 6.04 | 0.71 | (0.32, 1.59) | 0.4063 | 0.67 | (0.28, 1.62) | 0.3758 |
| Yes | 1166 | 89390 | 13.04 | 377 | 76614 | 4.92 | 0.4 | (0.36, 0.45) | <0.001 | 0.44 | (0.39, 0.49) | <0.001 |
| Sulfonylurea |  |  |  |  |  |  |  |  |  |  |  |  |
| No | 64 | 9557 | 6.70 | 37 | 9139 | 4.05 | 0.68 | (0.45, 1.03) | 0.0656 | 0.57 | (0.37, 0.86) | 0.0077 |
| Yes | 1120 | 81671 | 13.71 | 350 | 69130 | 5.06 | 0.4 | (0.35, 0.45) | <0.001 | 0.43 | (0.38, 0.48) | <0.001 |
| Meglitinides |  |  |  |  |  |  |  |  |  |  |  |  |
| No | 752 | 69225 | 10.86 | 242 | 60440 | 4.00 | 0.4 | (0.34, 0.46) | <0.001 | 0.41 | (0.35, 0.47) | <0.001 |
| Yes | 432 | 22003 | 19.63 | 145 | 17828 | 8.13 | 0.45 | (0.37, 0.55) | <0.001 | 0.5 | (0.41, 0.61) | <0.001 |
| Thiazolidinedione |  |  |  |  |  |  |  |  |  |  |  |  |
| No | 438 | 42100 | 10.40 | 154 | 37314 | 4.13 | 0.42 | (0.35, 0.51) | <0.001 | 0.44 | (0.36, 0.53) | <0.001 |
| Yes | 746 | 49128 | 15.18 | 233 | 40954 | 5.69 | 0.41 | (0.35, 0.47) | <0.001 | 0.44 | (0.38, 0.51) | <0.001 |
| DPP-4 inhibitors |  |  |  |  |  |  |  |  |  |  |  |  |
| No | 201 | 18371 | 10.94 | 69 | 15248 | 4.53 | 0.46 | (0.35, 0.61) | <0.001 | 0.47 | (0.36, 0.62) | <0.001 |
| Yes | 983 | 72857 | 13.49 | 318 | 63021 | 5.05 | 0.4 | (0.35, 0.45) | <0.001 | 0.43 | (0.38, 0.49) | <0.001 |
| SGLT2 inhibitors |  |  |  |  |  |  |  |  |  |  |  |  |
| No | 1157 | 81947 | 14.12 | 358 | 67829 | 5.28 | 0.4 | (0.35, 0.45) | <0.001 | 0.42 | (0.38, 0.48) | <0.001 |
| Yes | 27 | 9281 | 2.91 | 29 | 10440 | 2.78 | 0.96 | (0.57, 1.62) | 0.8682 | 0.91 | (0.54, 1.54) | 0.7237 |
| Insulin |  |  |  |  |  |  |  |  |  |  |  |  |
| No | 235 | 32317 | 7.27 | 68 | 28235 | 2.41 | 0.36 | (0.27, 0.47) | <0.001 | 0.37 | (0.28, 0.49) | <0.001 |
| Yes | 949 | 58912 | 16.11 | 319 | 50034 | 6.38 | 0.43 | (0.38, 0.48) | <0.001 | 0.45 | (0.4, 0.51) | <0.001 |
| ACEI/ARB |  |  |  |  |  |  |  |  |  |  |  |  |
| No | 186 | 28898 | 6.44 | 52 | 25340 | 2.05 | 0.34 | (0.25, 0.47) | <0.001 | 0.36 | (0.26, 0.49) | <0.001 |
| Yes | 998 | 62330 | 16.01 | 335 | 52928 | 6.33 | 0.43 | (0.38, 0.48) | <0.001 | 0.45 | (0.4, 0.51) | <0.001 |
| β-blockers |  |  |  |  |  |  |  |  |  |  |  |  |
| No | 972 | 74047 | 13.13 | 305 | 62975 | 4.84 | 0.4 | (0.35, 0.45) | <0.001 | 0.42 | (0.37, 0.48) | <0.001 |
| Yes | 212 | 17182 | 12.34 | 82 | 15294 | 5.36 | 0.47 | (0.37, 0.61) | <0.001 | 0.51 | (0.39, 0.66) | <0.001 |
| Calcium-channel blockers | |  |  |  |  |  |  |  |  |  |  |  |
| No | 339 | 44720 | 7.58 | 95 | 38336 | 2.48 | 0.35 | (0.28, 0.44) | <0.001 | 0.38 | (0.3, 0.47) | <0.001 |
| Yes | 845 | 46509 | 18.17 | 292 | 39933 | 7.31 | 0.43 | (0.38, 0.5) | <0.001 | 0.46 | (0.4, 0.53) | <0.001 |
| Diuretics |  |  |  |  |  |  |  |  |  |  |  |  |
| No | 539 | 60214 | 8.95 | 149 | 51274 | 2.91 | 0.36 | (0.3, 0.43) | <0.001 | 0.39 | (0.32, 0.46) | <0.001 |
| Yes | 645 | 31014 | 20.80 | 238 | 26995 | 8.82 | 0.45 | (0.39, 0.52) | <0.001 | 0.48 | (0.41, 0.56) | <0.001 |
| Statin |  |  |  |  |  |  |  |  |  |  |  |  |
| No | 261 | 20962 | 12.45 | 70 | 18824 | 3.72 | 0.32 | (0.24, 0.42) | <0.001 | 0.34 | (0.26, 0.44) | <0.001 |
| Yes | 923 | 70267 | 13.14 | 317 | 59444 | 5.33 | 0.44 | (0.39, 0.5) | <0.001 | 0.47 | (0.41, 0.53) | <0.001 |
| Aspirin |  |  |  |  |  |  |  |  |  |  |  |  |
| No | 548 | 57768 | 9.49 | 182 | 50426 | 3.61 | 0.41 | (0.35, 0.49) | <0.001 | 0.44 | (0.37, 0.52) | <0.001 |
| Yes | 636 | 33461 | 19.01 | 205 | 27842 | 7.36 | 0.42 | (0.36, 0.49) | <0.001 | 0.44 | (0.37, 0.51) | <0.001 |

*T2D* type 2 diabetes, *GLP-1 RAs* glucagon-like peptide-1 receptor agonists, *PY* person-years, *IR* incidence rate, per 1,000 person-years, *cHR* crude hazard ratio, *aHR* adjusted hazard ratio, *COPD* chronic obstructive pulmonary disease, *CCI* Charlson Comorbidity Index, *DCSI* Diabetes Complications Severity Index, *DPP-4* dipeptidyl peptidase-4, *SGLT2* sodium-glucose cotransporter-2, *ACEI* angiotensin-converting enzyme inhibitor, *ARB* angiotensin receptor blocker. aHR†: multivariable analysis, including sex, age, comorbidities, medications, CCI, DCSI, and number of oral antidiabetic drugs, as shown in Table 1

**Table S3** The risk of cardiovascular death for patients with and without GLP-1 RA stratified by variables

| **Variable** | **Non-GLP-1 RA** | | | **GLP-1 RA us** | | | **Crude** | | | **Adjusted†** | | |
| --- | --- | --- | --- | --- | --- | --- | --- | --- | --- | --- | --- | --- |
|  | **n** | **PY** | **IR** | **n** | **PY** | **IR** | **cHR** | **95% CI** | **P-value** | **aHR** | **95% CI** | **P-value** |
| Sex |  |  |  |  |  |  |  |  |  |  |  |  |
| female | 151 | 53631 | 2.82 | 53 | 46622 | 1.14 | 0.45 | (0.33, 0.62) | <0.001 | 0.53 | (0.39, 0.73) | <0.001 |
| male | 168 | 45138 | 3.72 | 50 | 38087 | 1.31 | 0.38 | (0.28, 0.52) | <0.001 | 0.41 | (0.3, 0.56) | <0.001 |
| Age |  |  |  |  |  |  |  |  |  |  |  |  |
| 20-40 | 21 | 17141 | 1.23 | 5 | 17478 | 0.29 | 0.23 | (0.09, 0.61) | 0.0032 | 0.22 | (0.08, 0.59) | 0.0028 |
| 41-60 | 119 | 50749 | 2.34 | 45 | 44056 | 1.02 | 0.48 | (0.34, 0.67) | <0.001 | 0.5 | (0.35, 0.7) | <0.001 |
| >60 | 179 | 30879 | 5.80 | 53 | 23175 | 2.29 | 0.47 | (0.34, 0.64) | <0.001 | 0.51 | (0.37, 0.69) | <0.001 |
| Comorbidities |  |  |  |  |  |  |  |  |  |  |  |  |
| Obesity |  |  |  |  |  |  |  |  |  |  |  |  |
| No | 304 | 89012 | 3.42 | 95 | 73748 | 1.29 | 0.41 | (0.33, 0.52) | <0.001 | 0.47 | (0.37, 0.59) | <0.001 |
| Yes | 15 | 9758 | 1.54 | 8 | 10961 | 0.73 | 0.48 | (0.2, 1.13) | 0.0913 | 0.5 | (0.21, 1.2) | 0.1202 |
| Smoking |  |  |  |  |  |  |  |  |  |  |  |  |
| No |  |  |  |  |  |  | 0.42 | (0.33, 0.52) | <0.001 | 0.48 | (0.38, 0.6) | <0.001 |
| Yes |  |  |  |  |  |  | 0.13 | (0.02, 1.05) | 0.0559 | 0.11 | (0.01, 0.96) | 0.0458 |
| Hypertension |  |  |  |  |  |  |  |  |  |  |  |  |
| No | 45 | 28664 | 1.57 | 8 | 25256 | 0.32 | 0.22 | (0.1, 0.47) | <0.001 | 0.25 | (0.12, 0.54) | <0.001 |
| Yes | 274 | 70105 | 3.91 | 95 | 59453 | 1.60 | 0.45 | (0.35, 0.56) | <0.001 | 0.5 | (0.4, 0.64) | <0.001 |
| Dyslipidemia |  |  |  |  |  |  |  |  |  |  |  |  |
| No | 58 | 14853 | 3.90 | 15 | 13601 | 1.10 | 0.3 | (0.17, 0.53) | <0.001 | 0.39 | (0.22, 0.69) | 0.0014 |
| Yes | 261 | 83916 | 3.11 | 88 | 71108 | 1.24 | 0.44 | (0.34, 0.56) | <0.001 | 0.49 | (0.38, 0.63) | <0.001 |
| Stroke |  |  |  |  |  |  |  |  |  |  |  |  |
| No | 278 | 94289 | 2.95 | 84 | 81286 | 1.03 | 0.38 | (0.3, 0.49) | <0.001 | 0.43 | (0.34, 0.55) | <0.001 |
| Yes | 41 | 4481 | 9.15 | 19 | 3424 | 5.55 | 0.7 | (0.4, 1.21) | 0.1997 | 0.72 | (0.41, 1.26) | 0.2494 |
| Coronary artery disease |  |  |  |  |  |  |  |  |  |  |  |  |
| No | 236 | 82383 | 2.86 | 82 | 71248 | 1.15 | 0.43 | (0.33, 0.56) | <0.001 | 0.5 | (0.39, 0.64) | <0.001 |
| Yes | 83 | 16387 | 5.07 | 21 | 13461 | 1.56 | 0.36 | (0.22, 0.59) | <0.001 | 0.37 | (0.23, 0.6) | <0.001 |
| Heart failure |  |  |  |  |  |  |  |  |  |  |  |  |
| No | 285 | 95257 | 2.99 | 95 | 81550 | 1.16 | 0.42 | (0.34, 0.54) | <0.001 | 0.49 | (0.39, 0.62) | <0.001 |
| Yes | 34 | 3512 | 9.68 | 8 | 3159 | 2.53 | 0.29 | (0.14, 0.64) | 0.0019 | 0.27 | (0.12, 0.6) | 0.0013 |
| Chronic kidney disease |  |  |  |  |  |  |  |  |  |  |  |  |
| No | 219 | 80714 | 2.71 | 67 | 70151 | 0.96 | 0.38 | (0.29, 0.51) | <0.001 | 0.44 | (0.33, 0.58) | <0.001 |
| Yes | 100 | 18056 | 5.54 | 36 | 14559 | 2.47 | 0.51 | (0.35, 0.75) | <0.001 | 0.55 | (0.37, 0.8) | 0.0022 |
| COPD |  |  |  |  |  |  |  |  |  |  |  |  |
| No | 240 | 76520 | 3.14 | 74 | 64862 | 1.14 | 0.4 | (0.3, 0.51) | <0.001 | 0.46 | (0.35, 0.6) | <0.001 |
| Yes | 79 | 22249 | 3.55 | 29 | 19848 | 1.46 | 0.45 | (0.29, 0.69) | <0.001 | 0.49 | (0.32, 0.76) | 0.0013 |
| CCI |  |  |  |  |  |  |  |  |  |  |  |  |
| 1 | 229 | 82485 | 2.78 | 77 | 70622 | 1.09 | 0.44 | (0.34, 0.56) | <0.001 | 0.49 | (0.38, 0.64) | <0.001 |
| 2-3 | 57 | 13662 | 4.17 | 20 | 11833 | 1.69 | 0.43 | (0.26, 0.72) | 0.0014 | 0.49 | (0.29, 0.82) | 0.007 |
| >3 | 33 | 2622 | 12.58 | 6 | 2254 | 2.66 | 0.24 | (0.1, 0.58) | 0.0015 | 0.24 | (0.1, 0.59) | 0.0018 |
| DCSI |  |  |  |  |  |  |  |  |  |  |  |  |
| 0 | 36 | 21543 | 1.67 | 14 | 19468 | 0.72 | 0.46 | (0.25, 0.86) | 0.0146 | 0.5 | (0.27, 0.94) | 0.0309 |
| 1 | 41 | 19722 | 2.08 | 12 | 17473 | 0.69 | 0.36 | (0.19, 0.69) | 0.0019 | 0.4 | (0.21, 0.76) | 0.0054 |
| ≥2 | 242 | 57504 | 4.21 | 77 | 47768 | 1.61 | 0.42 | (0.33, 0.55) | <0.001 | 0.47 | (0.37, 0.61) | <0.001 |
| Number of oral antidiabetic drugs | |  |  |  |  |  |  |  |  |  |  |  |
| ≦2 | 11 | 8994 | 1.22 | 5 | 7744 | 0.65 | 0.6 | (0.21, 1.77) | 0.357 | 0.63 | (0.21, 1.91) | 0.4153 |
| >2 | 308 | 89776 | 3.43 | 98 | 76965 | 1.27 | 0.4 | (0.32, 0.51) | <0.001 | 0.46 | (0.37, 0.58) | <0.001 |
| Medication |  |  |  |  |  |  |  |  |  |  |  |  |
| Metformin |  |  |  |  |  |  |  |  |  |  |  |  |
| No | 3 | 2026 | 1.48 | 4 | 1857 | 2.15 | 1.33 | (0.29, 6.06) | 0.7118 | 1.27 | (0.16, 10.16) | 0.8231 |
| Yes | 316 | 96744 | 3.27 | 99 | 82852 | 1.19 | 0.4 | (0.32, 0.5) | <0.001 | 0.46 | (0.36, 0.57) | <0.001 |
| Sulfonylurea |  |  |  |  |  |  |  |  |  |  |  |  |
| No | 17 | 9926 | 1.71 | 11 | 10053 | 1.09 | 0.7 | (0.32, 1.49) | 0.3535 | 0.58 | (0.27, 1.29) | 0.1821 |
| Yes | 302 | 88843 | 3.40 | 92 | 74656 | 1.23 | 0.4 | (0.31, 0.5) | <0.001 | 0.45 | (0.36, 0.57) | <0.001 |
| Meglitinides |  |  |  |  |  |  |  |  |  |  |  |  |
| No | 205 | 74984 | 2.73 | 61 | 65167 | 0.94 | 0.38 | (0.28, 0.5) | <0.001 | 0.41 | (0.31, 0.55) | <0.001 |
| Yes | 114 | 23786 | 4.79 | 42 | 19542 | 2.15 | 0.49 | (0.34, 0.7) | <0.001 | 0.57 | (0.4, 0.82) | 0.0022 |
| Thiazolidinedione |  |  |  |  |  |  |  |  |  |  |  |  |
| No | 119 | 45182 | 2.63 | 42 | 40317 | 1.04 | 0.43 | (0.3, 0.61) | <0.001 | 0.48 | (0.33, 0.68) | <0.001 |
| Yes | 200 | 53587 | 3.73 | 61 | 44392 | 1.37 | 0.4 | (0.3, 0.54) | <0.001 | 0.47 | (0.35, 0.63) | <0.001 |
| DPP-4 inhibitors |  |  |  |  |  |  |  |  |  |  |  |  |
| No | 73 | 19936 | 3.66 | 17 | 16482 | 1.03 | 0.31 | (0.18, 0.52) | <0.001 | 0.34 | (0.2, 0.58) | <0.001 |
| Yes | 246 | 78834 | 3.12 | 86 | 68227 | 1.26 | 0.44 | (0.34, 0.56) | <0.001 | 0.5 | (0.39, 0.65) | <0.001 |
| SGLT2 inhibitors |  |  |  |  |  |  |  |  |  |  |  |  |
| No | 312 | 88999 | 3.51 | 97 | 73364 | 1.32 | 0.4 | (0.32, 0.51) | <0.001 | 0.46 | (0.36, 0.58) | <0.001 |
| Yes | 7 | 9771 | 0.72 | 6 | 11345 | 0.53 | 0.78 | (0.26, 2.32) | 0.6497 | 0.65 | (0.21, 2.02) | 0.4566 |
| Insulin |  |  |  |  |  |  |  |  |  |  |  |  |
| No | 62 | 33855 | 1.83 | 21 | 29885 | 0.70 | 0.42 | (0.26, 0.69) | <0.001 | 0.48 | (0.29, 0.8) | 0.0044 |
| Yes | 257 | 64914 | 3.96 | 82 | 54824 | 1.50 | 0.41 | (0.32, 0.53) | <0.001 | 0.46 | (0.36, 0.6) | <0.001 |
| ACEI/ARB |  |  |  |  |  |  |  |  |  |  |  |  |
| No | 39 | 30759 | 1.27 | 14 | 27190 | 0.51 | 0.46 | (0.25, 0.84) | 0.0125 | 0.53 | (0.28, 0.98) | 0.0446 |
| Yes | 280 | 68010 | 4.12 | 89 | 57519 | 1.55 | 0.41 | (0.32, 0.52) | <0.001 | 0.46 | (0.36, 0.59) | <0.001 |
| β-blockers |  |  |  |  |  |  |  |  |  |  |  |  |
| No | 255 | 79335 | 3.21 | 83 | 67255 | 1.23 | 0.42 | (0.33, 0.54) | <0.001 | 0.48 | (0.37, 0.62) | <0.001 |
| Yes | 64 | 19434 | 3.29 | 20 | 17454 | 1.15 | 0.38 | (0.23, 0.63) | <0.001 | 0.43 | (0.26, 0.72) | 0.0013 |
| Calcium-channel blockers | |  |  |  |  |  |  |  |  |  |  |  |
| No | 82 | 47221 | 1.74 | 19 | 40791 | 0.47 | 0.29 | (0.18, 0.48) | <0.001 | 0.33 | (0.2, 0.54) | <0.001 |
| Yes | 237 | 51549 | 4.60 | 84 | 43918 | 1.91 | 0.46 | (0.36, 0.59) | <0.001 | 0.52 | (0.4, 0.67) | <0.001 |
| Diuretics |  |  |  |  |  |  |  |  |  |  |  |  |
| No | 128 | 63746 | 2.01 | 36 | 54545 | 0.66 | 0.36 | (0.25, 0.52) | <0.001 | 0.41 | (0.28, 0.6) | <0.001 |
| Yes | 191 | 35023 | 5.45 | 67 | 30164 | 2.22 | 0.45 | (0.34, 0.59) | <0.001 | 0.5 | (0.38, 0.66) | <0.001 |
| Statin |  |  |  |  |  |  |  |  |  |  |  |  |
| No | 67 | 22816 | 2.94 | 17 | 20308 | 0.84 | 0.3 | (0.18, 0.51) | <0.001 | 0.38 | (0.22, 0.66) | <0.001 |
| Yes | 252 | 75954 | 3.32 | 86 | 64401 | 1.34 | 0.44 | (0.35, 0.57) | <0.001 | 0.5 | (0.39, 0.63) | <0.001 |
| Aspirin |  |  |  |  |  |  |  |  |  |  |  |  |
| No | 138 | 61810 | 2.23 | 48 | 54363 | 0.88 | 0.43 | (0.31, 0.6) | <0.001 | 0.51 | (0.36, 0.71) | <0.001 |
| Yes | 181 | 36959 | 4.90 | 55 | 30346 | 1.81 | 0.41 | (0.3, 0.55) | <0.001 | 0.43 | (0.32, 0.59) | <0.001 |

*T2D* type 2 diabetes, *GLP-1 RAs* glucagon-like peptide-1 receptor agonists, *PY* person-years, *IR* incidence rate; per 1,000 person-years, *cHR* crude hazard ratio, aHR *adjusted hazard ratio*, *COPD* chronic obstructive pulmonary disease, *CCI* Charlson Comorbidity Index, *DCSI* Diabetes Complications Severity Index, *DPP-4* dipeptidyl peptidase-4, *SGLT2* sodium-glucose cotransporter-2, *ACEI* angiotensin-converting enzyme inhibitor, *ARB* angiotensin receptor blocker. aHR†: multivariable analysis, including sex, age, comorbidities, medications, CCI, DCSI, and number of oral antidiabetic drugs, as shown in Table 1. The blank spaces are due to values less than 4, and the data cannot be carried out from the Data Science Center to protect individual privacy

**Table S4** The risk of cardiovascular events for patients with and without GLP-1 RA stratified by variables

| **Variable** | **Non-GLP-1 RA** | | | **GLP-1 RA use** | | | **Crude** | | | **Adjusted** | | |
| --- | --- | --- | --- | --- | --- | --- | --- | --- | --- | --- | --- | --- |
|  | **n** | **PY** | **IR** | **n** | **PY** | **IR** | **cHR** | **95%CI** | **P-value** | **aHR** | **95%CI** | **P-value** |
| Sex |  |  |  |  |  |  |  |  |  |  |  |  |
| female | 1072 | 51534 | 20.80 | 719 | 45380 | 15.84 | 0.79 | (0.72, 0.87) | <0.001 | 0.89 | (0.81, 0.98) | 0.0151 |
| male | 1140 | 42939 | 26.55 | 767 | 36860 | 20.81 | 0.81 | (0.74, 0.89) | <0.001 | 0.85 | (0.77, 0.93) | <0.001 |
| Age |  |  |  |  |  |  |  |  |  |  |  |  |
| 20-40 | 135 | 16884 | 8.00 | 111 | 17279 | 6.42 | 0.81 | (0.63, 1.05) | 0.1095 | 0.82 | (0.63, 1.06) | 0.1224 |
| 41-60 | 919 | 48788 | 18.84 | 723 | 42791 | 16.90 | 0.93 | (0.84, 1.03) | 0.1578 | 0.96 | (0.87, 1.05) | 0.3666 |
| >60 | 1158 | 28801 | 40.21 | 652 | 22170 | 29.41 | 0.8 | (0.72, 0.88) | <0.001 | 0.81 | (0.73, 0.89) | <0.001 |
| Comorbidities |  |  |  |  |  |  |  |  |  |  |  |  |
| Obesity |  |  |  |  |  |  |  |  |  |  |  |  |
| No | 2075 | 84939 | 24.43 | 1327 | 71609 | 18.53 | 0.79 | (0.74, 0.85) | <0.001 | 0.85 | (0.79, 0.91) | <0.001 |
| Yes | 137 | 9535 | 14.37 | 159 | 10631 | 14.96 | 1.04 | (0.83, 1.31) | 0.7427 | 1.08 | (0.85, 1.35) | 0.5391 |
| Smoking |  |  |  |  |  |  |  |  |  |  |  |  |
| No | 2145 | 91618 | 23.41 | 1430 | 79369 | 18.02 | 0.8 | (0.75, 0.85) | <0.001 | 0.86 | (0.81, 0.93) | <0.001 |
| Yes | 67 | 2856 | 23.46 | 56 | 2871 | 19.50 | 0.84 | (0.59, 1.2) | 0.3403 | 0.88 | (0.61, 1.26) | 0.4894 |
| Hypertension |  |  |  |  |  |  |  |  |  |  |  |  |
| No | 329 | 28014 | 11.74 | 200 | 24881 | 8.04 | 0.73 | (0.61, 0.87) | <0.001 | 0.79 | (0.66, 0.95) | 0.0101 |
| Yes | 1883 | 66459 | 28.33 | 1286 | 57358 | 22.42 | 0.82 | (0.76, 0.88) | <0.001 | 0.88 | (0.82, 0.94) | <0.001 |
| Dyslipidemia |  |  |  |  |  |  |  |  |  |  |  |  |
| No | 321 | 14188 | 22.63 | 203 | 13181 | 15.40 | 0.69 | (0.58, 0.83) | <0.001 | 0.8 | (0.67, 0.96) | 0.0139 |
| Yes | 1891 | 80286 | 23.55 | 1283 | 69059 | 18.58 | 0.82 | (0.77, 0.88) | <0.001 | 0.88 | (0.82, 0.95) | <0.001 |
| Stroke |  |  |  |  |  |  |  |  |  |  |  |  |
| No | 1979 | 90427 | 21.89 | 1342 | 79034 | 16.98 | 0.81 | (0.75, 0.86) | <0.001 | 0.87 | (0.81, 0.93) | <0.001 |
| Yes | 233 | 4046 | 57.58 | 144 | 3205 | 44.92 | 0.83 | (0.68, 1.03) | 0.0898 | 0.89 | (0.72, 1.11) | 0.298 |
| Coronary artery disease |  |  |  |  |  |  |  |  |  |  |  |  |
| No | 1531 | 79476 | 19.26 | 1003 | 69604 | 14.41 | 0.78 | (0.72, 0.84) | <0.001 | 0.86 | (0.79, 0.93) | <0.001 |
| Yes | 681 | 14997 | 45.41 | 483 | 12636 | 38.23 | 0.88 | (0.78, 0.99) | 0.0371 | 0.9 | (0.8, 1.01) | 0.0716 |
| Heart failure |  |  |  |  |  |  |  |  |  |  |  |  |
| No | 2029 | 91276 | 22.23 | 1356 | 79301 | 17.10 | 0.8 | (0.75, 0.86) | <0.001 | 0.87 | (0.81, 0.93) | <0.001 |
| Yes | 183 | 3197 | 57.23 | 130 | 2938 | 44.24 | 0.8 | (0.63, 1) | 0.0466 | 0.82 | (0.66, 1.04) | 0.0998 |
| Chronic kidney disease |  |  |  |  |  |  |  |  |  |  |  |  |
| No | 1548 | 77502 | 19.97 | 1051 | 68249 | 15.40 | 0.8 | (0.74, 0.87) | <0.001 | 0.87 | (0.8, 0.94) | <0.001 |
| Yes | 664 | 16971 | 39.13 | 435 | 13991 | 31.09 | 0.84 | (0.74, 0.95) | 0.0052 | 0.86 | (0.76, 0.98) | 0.0188 |
| COPD |  |  |  |  |  |  |  |  |  |  |  |  |
| No | 1574 | 73374 | 21.45 | 1065 | 63091 | 16.88 | 0.82 | (0.75, 0.88) | <0.001 | 0.89 | (0.83, 0.97) | 0.005 |
| Yes | 638 | 21099 | 30.24 | 421 | 19149 | 21.99 | 0.76 | (0.67, 0.86) | <0.001 | 0.81 | (0.71, 0.92) | <0.001 |
| CCI |  |  |  |  |  |  |  |  |  |  |  |  |
| 1 | 1644 | 79095 | 20.79 | 1103 | 68711 | 16.05 | 0.8 | (0.75, 0.87) | <0.001 | 0.88 | (0.82, 0.95) | 0.0014 |
| 2-3 | 421 | 12964 | 32.48 | 300 | 11357 | 26.41 | 0.85 | (0.73, 0.99) | 0.035 | 0.89 | (0.77, 1.04) | 0.1353 |
| >3 | 147 | 2415 | 60.88 | 83 | 2171 | 38.23 | 0.66 | (0.5, 0.87) | 0.0027 | 0.66 | (0.5, 0.87) | 0.0036 |
| DCSI |  |  |  |  |  |  |  |  |  |  |  |  |
| 0 | 229 | 21067 | 10.87 | 147 | 19184 | 7.66 | 0.76 | (0.61, 0.93) | 0.0088 | 0.81 | (0.66, 1) | 0.0479 |
| 1 | 297 | 18999 | 15.63 | 210 | 17098 | 12.28 | 0.82 | (0.69, 0.98) | 0.0315 | 0.89 | (0.75, 1.07) | 0.2175 |
| ≥2 | 1686 | 54408 | 30.99 | 1129 | 45958 | 24.57 | 0.83 | (0.77, 0.89) | <0.001 | 0.87 | (0.8, 0.94) | <0.001 |
| Number of oral antidiabetic drugs | |  |  |  |  |  |  |  |  |  |  |  |
| 1 | 37 | 2894 | 12.78 | 21 | 2521 | 8.33 | 0.77 | (0.45, 1.34) | 0.3585 | 0.94 | (0.51, 1.72) | 0.8411 |
| 2-3 | 336 | 21213 | 15.84 | 241 | 19477 | 12.37 | 0.82 | (0.7, 0.97) | 0.0208 | 0.88 | (0.74, 1.04) | 0.1217 |
| >3 | 1839 | 70366 | 26.13 | 1224 | 60242 | 20.32 | 0.81 | (0.75, 0.87) | <0.001 | 0.87 | (0.81, 0.93) | <0.001 |
| Medication |  |  |  |  |  |  |  |  |  |  |  |  |
| Metformin |  |  |  |  |  |  |  |  |  |  |  |  |
| No | 56 | 1888 | 29.66 | 25 | 1821 | 13.73 | 0.52 | (0.32, 0.83) | 0.0067 | 0.54 | (0.32, 0.9) | 0.0191 |
| Yes | 2156 | 92585 | 23.29 | 1461 | 80419 | 18.17 | 0.81 | (0.76, 0.86) | <0.001 | 0.88 | (0.82, 0.94) | <0.001 |
| Sulfonylurea |  |  |  |  |  |  |  |  |  |  |  |  |
| No | 122 | 9678 | 12.61 | 131 | 9851 | 13.30 | 1.1 | (0.86, 1.41) | 0.4582 | 1 | (0.77, 1.28) | 0.9746 |
| Yes | 2090 | 84795 | 24.65 | 1355 | 72389 | 18.72 | 0.79 | (0.74, 0.84) | <0.001 | 0.85 | (0.8, 0.91) | <0.001 |
| Meglitinides |  |  |  |  |  |  |  |  |  |  |  |  |
| No | 1511 | 71913 | 21.01 | 1051 | 63350 | 16.59 | 0.82 | (0.76, 0.89) | <0.001 | 0.88 | (0.81, 0.95) | 0.0012 |
| Yes | 701 | 22561 | 31.07 | 435 | 18890 | 23.03 | 0.77 | (0.68, 0.87) | <0.001 | 0.84 | (0.75, 0.95) | 0.0059 |
| Thiazolidinedione |  |  |  |  |  |  |  |  |  |  |  |  |
| No | 834 | 43623 | 19.12 | 622 | 39286 | 15.83 | 0.86 | (0.78, 0.96) | 0.0064 | 0.95 | (0.85, 1.05) | 0.3219 |
| Yes | 1378 | 50851 | 27.10 | 864 | 42953 | 20.11 | 0.77 | (0.7, 0.84) | <0.001 | 0.82 | (0.75, 0.9) | <0.001 |
| DPP-4 inhibitors |  |  |  |  |  |  |  |  |  |  |  |  |
| No | 362 | 19179 | 18.87 | 240 | 16069 | 14.94 | 0.86 | (0.73, 1.01) | 0.0646 | 0.93 | (0.78, 1.1) | 0.3851 |
| Yes | 1850 | 75294 | 24.57 | 1246 | 66171 | 18.83 | 0.79 | (0.73, 0.85) | <0.001 | 0.86 | (0.8, 0.92) | <0.001 |
| SGLT2 inhibitors |  |  |  |  |  |  |  |  |  |  |  |  |
| No | 2100 | 84784 | 24.77 | 1335 | 71015 | 18.80 | 0.78 | (0.73, 0.84) | <0.001 | 0.85 | (0.8, 0.91) | <0.001 |
| Yes | 112 | 9689 | 11.56 | 151 | 11225 | 13.45 | 1.14 | (0.89, 1.46) | 0.2889 | 1.05 | (0.82, 1.35) | 0.6765 |
| Insulin |  |  |  |  |  |  |  |  |  |  |  |  |
| No | 507 | 32735 | 15.49 | 359 | 29268 | 12.27 | 0.83 | (0.73, 0.96) | 0.0092 | 0.91 | (0.79, 1.04) | 0.1554 |
| Yes | 1705 | 61738 | 27.62 | 1127 | 52972 | 21.28 | 0.8 | (0.74, 0.86) | <0.001 | 0.85 | (0.79, 0.92) | <0.001 |
| ACEI/ARB |  |  |  |  |  |  |  |  |  |  |  |  |
| No | 353 | 30089 | 11.73 | 237 | 26757 | 8.86 | 0.81 | (0.68, 0.95) | 0.012 | 0.87 | (0.74, 1.03) | 0.1081 |
| Yes | 1859 | 64385 | 28.87 | 1249 | 55483 | 22.51 | 0.81 | (0.75, 0.87) | <0.001 | 0.86 | (0.8, 0.93) | <0.001 |
| β-blockers |  |  |  |  |  |  |  |  |  |  |  |  |
| No | 1722 | 75998 | 22.66 | 1106 | 65411 | 16.91 | 0.78 | (0.72, 0.84) | <0.001 | 0.84 | (0.77, 0.9) | <0.001 |
| Yes | 490 | 18476 | 26.52 | 380 | 16828 | 22.58 | 0.88 | (0.77, 1.01) | 0.0684 | 0.97 | (0.84, 1.11) | 0.6237 |
| Calcium-channel blockers | |  |  |  |  |  |  |  |  |  |  |  |
| No | 637 | 45846 | 13.89 | 431 | 40048 | 10.76 | 0.82 | (0.72, 0.92) | 0.0012 | 0.89 | (0.79, 1.01) | 0.0697 |
| Yes | 1575 | 48627 | 32.39 | 1055 | 42192 | 25.01 | 0.8 | (0.74, 0.87) | <0.001 | 0.86 | (0.79, 0.93) | <0.001 |
| Diuretics |  |  |  |  |  |  |  |  |  |  |  |  |
| No | 1031 | 61613 | 16.73 | 684 | 53358 | 12.82 | 0.8 | (0.73, 0.89) | <0.001 | 0.88 | (0.8, 0.97) | 0.0124 |
| Yes | 1181 | 32861 | 35.94 | 802 | 28882 | 27.77 | 0.8 | (0.73, 0.87) | <0.001 | 0.86 | (0.78, 0.94) | <0.001 |
| Statin |  |  |  |  |  |  |  |  |  |  |  |  |
| No | 386 | 22037 | 17.52 | 272 | 19785 | 13.75 | 0.8 | (0.69, 0.94) | 0.0054 | 0.88 | (0.75, 1.03) | 0.1237 |
| Yes | 1826 | 72436 | 25.21 | 1214 | 62454 | 19.44 | 0.8 | (0.75, 0.87) | <0.001 | 0.86 | (0.8, 0.93) | <0.001 |
| Aspirin |  |  |  |  |  |  |  |  |  |  |  |  |
| No | 1007 | 59949 | 16.80 | 637 | 53356 | 11.94 | 0.74 | (0.67, 0.82) | <0.001 | 0.82 | (0.74, 0.91) | <0.001 |
| Yes | 1205 | 34524 | 34.90 | 849 | 28884 | 29.39 | 0.88 | (0.8, 0.96) | 0.0032 | 0.91 | (0.83, 0.99) | 0.0289 |

*T2D* type 2 diabetes, *GLP-1 RAs* glucagon-like peptide-1 receptor agonists, *PY* person-years, *IR* incidence rate, per 1,000 person-years, *cHR* crude hazard ratio, *aHR* adjusted hazard ratio, *COPD* chronic obstructive pulmonary disease, *CCI* Charlson Comorbidity Index, *DCSI* Diabetes Complications Severity Index, *DPP-4* dipeptidyl peptidase-4, *SGLT2* sodium-glucose cotransporter-2, *ACEI* angiotensin-converting enzyme inhibitor, *ARB* angiotensin receptor blocker. aHR†: multivariable analysis, including sex, age, comorbidities, medications, CCI, DCSI, and number of oral antidiabetic drugs, as shown in Table 1

**Table S5** The risk of liver-related death for patients with and without GLP-1 RA stratified by variables

| **Variable** | **Non-GLP-1 RA** | | | **GLP-1 RA use** | | | **Crude** | | | **Adjusted†** | | |
| --- | --- | --- | --- | --- | --- | --- | --- | --- | --- | --- | --- | --- |
|  | **n** | **PY** | **IR** | **n** | **PY** | **IR** | **cHR** | **95%CI** | **P-value** | **aHR** | **95% CI** | **P-value** |
| Sex |  |  |  |  |  |  |  |  |  |  |  |  |
| female | 15 | 53631 | 0.28 | 4 | 46622 | 0.09 | 0.3 | (0.1, 0.89) | 0.0308 | 0.32 | (0.11, 0.97) | 0.0441 |
| male | 20 | 45138 | 0.44 | 3 | 38087 | 0.08 | 0.21 | (0.06, 0.72) | 0.0126 | 0.23 | (0.07, 0.79) | 0.0192 |
| Age |  |  |  |  |  |  |  |  |  |  |  |  |
| <70 | 26 | 89372 | 0.29 | 3 | 78079 | 0.04 | 0.14 | (0.04, 0.47) | 0.0014 | 0.14 | (0.04, 0.47) | 0.0015 |
| ≧70 | 9 | 9397 | 0.96 | 4 | 6630 | 0.60 | 0.76 | (0.23, 2.52) | 0.6486 | 0.82 | (0.24, 2.81) | 0.7496 |

*T2D* type 2 diabetes, *GLP-1 RAs* glucagon-like peptide-1 receptor agonists, *PY* person-years, *IR* incidence rate, per 1,000 person-years, *cHR* crude hazard ratio, *aHR* adjusted hazard ratio, aHR†: multivariable analysis, including sex, age, comorbidities, medications, CCI, DCSI, and number of oral antidiabetic drugs, as shown in Table 1

**Figure captions**

**Fig. S1** Flowchart of patient selection in this study

**Fig. S2** Cumulative incidences of MACE (**a**), cardiovascular death (**b**), between GLP-1 RA users and nonusers. MACE, adverse cardiovascular events.
