# Supplementary figures and images for: Effects of glucagon-like peptide-1 receptor agonists on liver-related and cardiovascular mortality in patients with type 2 diabetes
Source: BMC Med. 2024 Jan 4;22:8. doi: 10.1186/s12916-023-03228-4 (PMC10765623; doi:10.1186/s12916-023-03228-4)

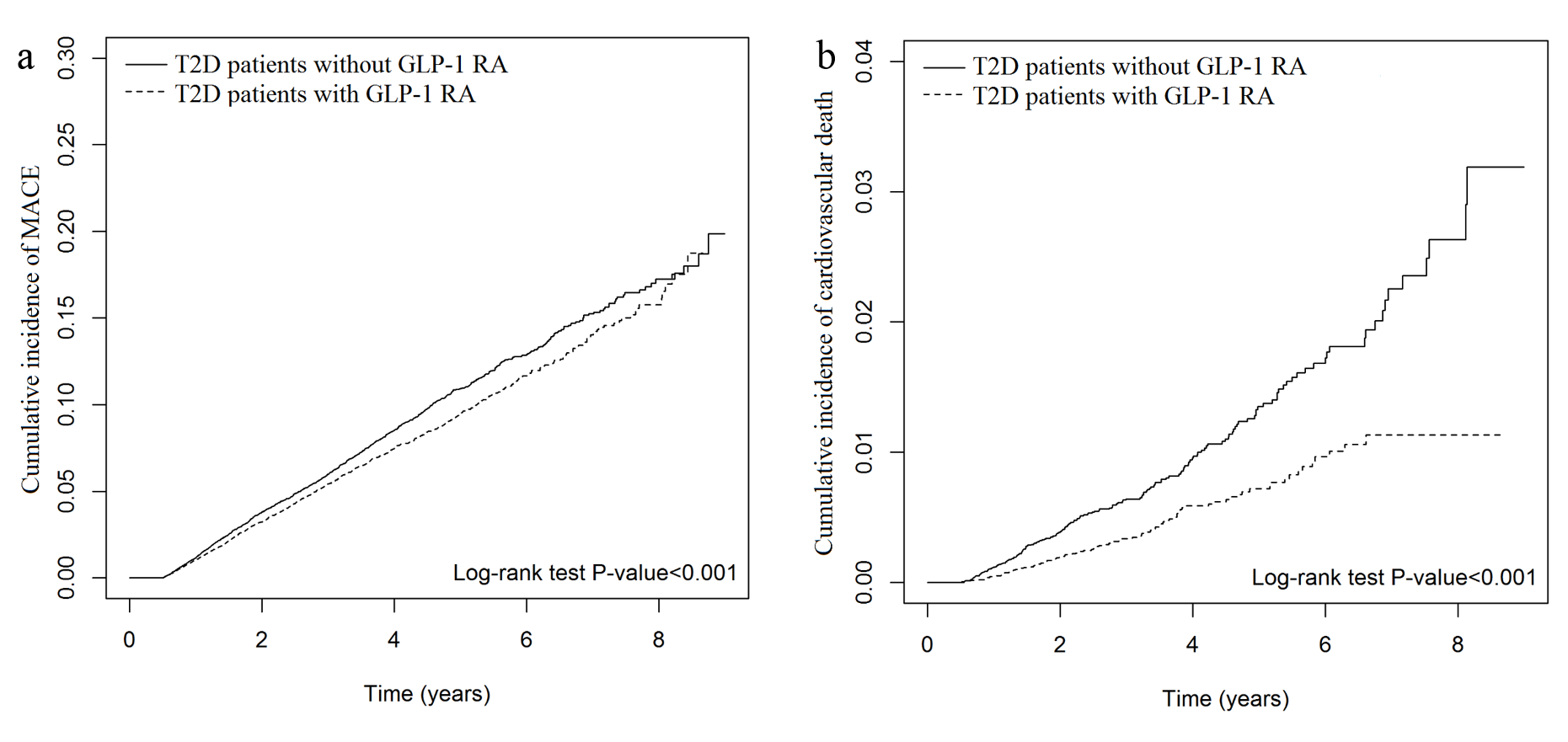

Supplement: Supplementary file 1 — Additional file 1: Table S1. Diseases and related ICD-9-CM, ICD-10-CM codes. Table S2. The risk of all-cause death for T2D patients with and without GLP-1 RA stratified by variables. Table S3. The risk of cardiovascular death for T2D patients with and without GLP-1 RA stratified by variables. Table S4. The risk of cardiovascular events for T2D patients with and without GLP-1 RA stratified by variables. Table S5. The risk of liver-related death for T2D patients with and without GLP-1 RA stratified by variables. Fig. S1. Flowchart of patient selection in this study. Fig. S2. The cumulative incidences of major adverse cardiovascular events (MACE, a), cardiovascular death (b), between GLP-1 RA users and nonusers in persons with T2D. [file 12916_2023_3228_MOESM1_ESM.zip › Fig. S2_formattedR3.tiff]
